# Lord Lister: VII. Antisepis and Asepsis

**Published:** 1918-03-09

**Authors:** 


### LORD LISTER.

### VII.—Antisepsis and Asepsis.

THE immense help and stimulus that Lister received from the researches of Pasteur will now be evident. Lister was already convinced that not only hospital diseases, but also suppuration itself, is due to the access to the wound of something in the air; he now learned from Pasteur what that something is, or, at any rate, what it is likely to be. He learned that it consists of solid particles, and that these solid particles can be filtered out of the air. He learned that these solid particles are living particles, and produce their baneful effects by a vital process; and that these living particles can be killed and thus prevented from producing any effect. It was on these principles that his practice was henceforth to be founded; but he still attached an importance that we now know to be exaggerated to the air as a medium of contamination, and he did not yet realise the paramount importance of the surgeon's hands and of his instruments in carrying the contagion. He did, indeed, recognise that the hands and instruments of the surgeon might carry the contagion, but he attached paramount importance to the air-borne germs, and it was some years before the parts were reversed in his estimation, and the importance of the air as a carrier relegated to a position below that of solids and liquids. Hence it happened that for years attention, energy, and industry were concentrated upon endeavours to sterilise the air, and the sterilisation of the solids and liquids that came in contact with the wound—the surgeon's hands and instruments—was comparatively, though by no means wholly neglected.

Lister called his system "the antiseptic system," which it was in part, and there are still people so ignorant as to suppose that asepsis is an improvement on the antiseptic system of Lister, or a wholly independent system, and that it was invented and adopted without Lister's knowledge, or even in spite of his opposition. The truth is, of course, that Lister's system was from the very first both antiseptic and aseptic, but was mainly and especially aseptic. The difference is this: that antisepsis seeks to destroy the putrefactive, purigenous, and pathogenic agents that have already gained access to a wound, while asepsis seeks to prevent these agents from gaining access to the wound. The one system is the complement of the other, and no one who understands and grasps the doctrine of the infectivity of wounds could possibly propose to employ the one without the other. When the surgeon makes a wound in the hitherto unbroken skin, as he must do at the beginning of many operations, he employs the aseptic system, for there are in the healthy tissues beneath the skin no pathogenic agents, or none of which he need take account, and all that is necessary is to prevent any such agents from gaining access to the wound; but when the wound has been already made before the surgeon sees the case, when the wound is the result of an accident, and the raw surfaces have already been contaminated, or have had the chance of being contaminated, by the lacerating or crushing agent, or by contact with the clothes, the earth, or other contaminating agents, even the air, then the mere exclusion of additional pathogenic germs is futile. Then it is not enough to prevent further contamination. Then, before the aseptic method can profitably be brought into play, the germs that have already gained access to the wound must be destroyed or must be deprived of their infective property, and this purification of an already infected wound is what we now mean by antisepsis as distinguished from asepsis, or the exclusion of additional germs. It is ridiculous in the extreme to suppose that a man of Lister's calibre did not recognise that asepsis is the complement of antisepsis, or that he would employ antisepsis to the neglect of asepsis; and, in fact, his teaching from the very first included both systems, but he included both under the name of antisepsis, considering, as is evident to the most rudimentary intelligence, that both are founded on the same principle, and that both or only one needs to be employed according to the nature of the case. After his system of treatment had been in operation for some years, the two applications of the principle were formally distinguished by the application to them of distinguishing names. One was called antisepsis, and the other was called asepsis; and ignorant people, misled, as ignorant people always are, by names, supposed that since Lister called his system antiseptic, therefore he employed only that half that we now call antisepsis, and neglected the other half that we now call asepsis. A more stupid instance of the vulgar error of mistaking names for things it would be difficult to find, but an equally stupid instance is afforded by the history of the reception of Lister's doctrine.

Lister attached, as we have seen, paramount and even exaggerated importance to the agency of the air as a carrier of infection, and he set himself to put a stop to the infection of wounds by the air-borne microbes. There are four conceivable methods of doing this. If it were possible, the air might be excluded altogether from the wound. If this is impracticable, as it is, then the air must be sterilised, and this can be done in either of four ways. The pathogenic germs may be filtered out from the air that gains access to the wound, or they may be destroyed; and if destroyed, they may be destroyed either by heat or by some chemical agent. There is yet a fifth method, by which, though the microbes are not actually killed, they are yet rendered inert and innocuous, but this is really a matter of detail, since the same agents that kill them serve, in milder application, to render them inert. Of these methods, Lister chose that of safeguarding the wound by enveloping it in material saturated with a chemical germicide through which no living germ could pass. This was very well for a beginning, and was the foundation of his treatment; but it was manifestly only a beginning, for

☞ Previous articles appeared Jan 26, Feb 2, 9, 16, 23, and March 2, p. 457.

**LORD LISTER**—(*continued*).

there were yet two contingencies unprovided for. The first of these was the contamination of an accidental wound before it came under the hands of the surgeon, and the second was the probability that when discharges escaped from the wound and penetrated the dressing, the track of the discharge would furnish a means of direct access for decomposition to reach the wound itself. The part of the discharge that had escaped beyond the dressing would decompose, and the decomposition would travel along the discharge to the wound itself. Hence it was necessary, in the first place, to cleanse the accidental wound thoroughly, and to treat its surface and all its cavities, pockets, and crevices with germicide; and, in the second place, to devise some means of preventing the spread of decomposition along the track of the discharge.

When Lister first arrived at these conclusions, about the year 1865, chemistry was in a very rudimentary stage compared with what it now is; and a satisfactory germicide was hard to find. Lister ultimately chose carbolic acid, as being the most powerful disinfectant then known, and by another stupidity, his system became identified with the use of carbolic acid, the principle on which its employment was founded was ignored, the reasoning that induced him to employ it received no attention, and Listerism was regarded in London as nothing but the substitution of a dressing of carbolic acid for the dressings then in vogue, such as poultices, water dressing, lead lotion, and so forth. His treatment was called "the carbolic acid treatment"; those who wished to make a trial of it, and had not been brought up under his own tuition, thought that if they dressed a wound with lint soaked in a solution of carbolic acid they were employing Lister's methods, and as their results were no better than they had been before, Listerism was discredited, and declared to be no improvement on the older methods.

The remaining history of Listerism is a history of constant experimentation and gradual approach to completeness in the attainment of these two ends —the purification of the wound from contamination suffered previous to the manipulations of the surgeon, and the prevention of contamination after it had come under his care or been inflicted by him. It is wonderful to contemplate the number of experiments that Lister conducted, and the care and assiduity that he bestowed upon them through many years, during which he was building up and maintaining the greatest surgical clinic in Europe, and doing an extensive private practice in addition. It is difficult for those who know only the present practice of surgery, and know nothing of what surgery was before Listerism, to appreciate the arduousness of his task. First an appropriate chemical germicide was to be found. Carbolic acid was effectual, but it had many disadvantages. When applied to the surfaces of the wounds it killed not only the germs in the wound, but also the healthy cells on the surface. It sometimes produced carbolic acid poisoning, by absorption from the cut or lacerated surface. It irritated the skin around the wound, and made it sore. Its caustic action on the surgeon's skin was very unpleasant, and blunted his delicate sense of touch; and when the carbolic spray was introduced, the inhalation of the acid irritated the bronchial tubes and lungs of patient, operator, assistants, and spectators. Clearly, it could be employed only until something better could be found; and everything that was without some of the disadvantages of carbolic acid had other disadvantages of its own.

(*To be continued.*)

